# The Diffusion of Intravenously Administered Cefuroxime and Metronidazole into the Peritoneal Fluid During Postoperative Period in Patients with Secondary Peritonitis Compared to Controls: A Pilot Study Using Peritoneal Microdialysis

**DOI:** 10.3390/antibiotics15040340

**Published:** 2026-03-26

**Authors:** Kristine Jung, Mark Bremholm Ellebæk, Per Damkier, Palle B. N. Fruekilde, Sören Möller, Ester Maria Gill, Jonas Emil Sabroe, Anne Riis Axelsen, Niels Qvist

**Affiliations:** 1 Research Unit for Surgery, Odense University Hospital, University of Southern Denmark, 5230 Odense, Denmark; kristinejung@hotmail.com (K.J.); mark.ellebaek1@rsyd.dk (M.B.E.); ester.maria.gill@rsyd.dk (E.M.G.); anne.axelsen@rsyd.dk (A.R.A.); 2 Research Unit for Clinical Pharmacology, Odense University Hospital, University of Southern Denmark, 5230 Odense, Denmark; per.damkier@rsyd.dk; 3 Department of Clinical biochemistry, Odense University Hospital, 5000 Odense, Denmark; 4 Research Unit for Epidemiology, Biostatistics and Biodemography, Department of Public Health, University of Southern Denmark, 5230 Odense, Denmark; moeller@health.sdu.dk

**Keywords:** cefuroxime, metronidazole, microdialysis, peritoneal fluid, secondary peritonitis, inflammatory bowel disease

## Abstract

**Background/Objectives:** To prevent surgical site infections, it is important to consider the concentration of the administered antibiotic in the target compartment. We measured the concentrations of cefuroxime and metronidazole in peritoneal fluid with the microdialysis technique in patients undergoing surgery for secondary peritonitis (7 patients) and for inflammatory bowel disease (11 patients). **Methods:** All patients received 1.5 g of cefuroxime and 0.5 g of metronidazole every 8 h during the postoperative period for at least 72 h. Microdialysates covering 8-h intervals were collected, and the concentration of cefuroxime and metronidazole was measured using liquid chromatography–mass spectrometry. **Results:** For metronidazole, a concentration of ≥4 μg/mL was reached in all but one sample, corresponding to the minimal inhibitory concentration (MIC) for most anaerobic bacteria strains. For cefuroxime, a value of ≥4 μg/mL was reached in 88% and 93% of the samples in the peritonitis group and the IBD group, respectively, corresponding to the MIC values for most Gram-negative bacteria, and a value of ≥16 μg/mL, corresponding to the MIC value for more resistant bacteria, was reached in only 40% and 23% of the samples, respectively. **Conclusions:** Our results show that the peritoneal microdialysis method is feasible for studying the diffusion of antibiotics into the peritoneal cavity. Measuring the accumulative concentration of antibiotics in the peritoneal fluid corresponding to the drug administration interval may provide important information to consider alongside traditional pharmacodynamic parameters and may be relevant to achieving an optimal therapeutic effect.

## 1. Introduction

Surgical site infections (SSIs) have a great impact on postoperative morbidity and hospital stay and constitute a major economic burden on health care systems [1]. Abdominal surgery carries the highest risk compared to other types of surgery, with an incidence between 4.1% and 26.7% [2]. The main factors used to determine this variability include underlying disease, type of intervention, comorbidity, and immunosuppressive treatment. Factors such as surveillance and data collection method also play an important role.

Several regimens have been recommended for antibiotic treatment in surgery for secondary peritonitis and for prophylaxis in elective abdominal surgery. At our institution, the combination of metronidazole and cefuroxime is a standard regime. The treatment goal should be to achieve concentrations above the minimum inhibitory concentration (MIC) of various bacterial strains in the peritoneal cavity during the treatment period. Studies suggest that a concentration of antibiotics above a given MIC value for at least 40–50% of the time (T > MIC) within the dosing interval results in adequate clinical and microbiological efficacy [3]. MIC values for metronidazole against anaerobe species vary considerably [4], with a breakpoint of 4 μg/mL according to The European Committee on Antimicrobial Susceptibility Testing (EUCAST) [5]. For cefuroxime, the MIC value for Gram-negative bacteria for most species is 4 μg/mL and it is higher for more resistant *Escherichia coli* strains, with a confidence interval of 8–16 μg/mL [5].

The diffusion of antibiotics into the peritoneal fluid is an important issue in both the prophylaxis of surgical site infections in elective abdominal surgery and in surgery for secondary peritonitis. An inflamed peritoneal surface may influence the capability of antibiotics to pass through the peritoneal lining into the peritoneal cavity, which might have an important clinical implication in relation to dosing regimens to achieve optimal treatment results without a risk of overdosing. To the best of our knowledge, this has not been investigated previously in patients undergoing acute surgery for secondary peritonitis or in elective surgery. Microdialysis is a known and well-established principle for measuring the concentration of antibiotics in different tissue compartments [6].

The primary objective of this study was to investigate whether the concentrations of cefuroxime and metronidazole reached the MIC values for Gram-negative bacteria, *Eschericia coli*, and anaerobic bacteria, respectively, in the peritoneal cavity in patients undergoing either emergency surgery for secondary diffuse peritonitis or elective surgery for inflammatory bowel disease (IBD). The secondary objective was to investigate whether the sampling period in relation to antibiotic administration had influence on the results.

## 2. Results

The distribution of age, gender, and body mass index (BMI) in each group is shown in Table 1. The rate of missing samples in the IBD group was 5.6% for both metronidazole and cefuroxime, and 18% and 24% in the peritonitis group, respectively. Only 4 out of 7 participants in the peritonitis group and 5 out of 11 participants in the IBD group had a full data set for the collection of dialysates. The reasons for missing data were mainly due to errors during sample collection. One sample was lost during analysis. Two participants did not complete the study period: one due to skin irritation around the microdialysis catheter that was removed prematurely and one due to the catheter falling out. For some patients, the microdialysis period extended for more than the planned 72 h (8th interval), for a maximum of 12 intervals for cefuroxime and 13 for metronidazole. No participants experienced surgical site infectious or other complications related to the microdialysis catheter, and all patients experienced an uncomplicated postoperative course.

**Figure 1:**
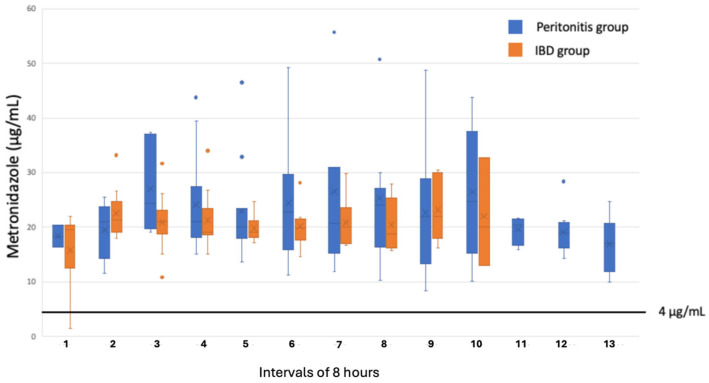
Box plots of concentrations of metronidazole in microdialysates collected within 8 h from the peritoneal cavity in patients electively operated on for inflammatory bowel disease (IBD) and patients operated on for acute secondary peritonitis. Boxes indicate the interquartile rage (IQR) for Q1–Q3; bars, the maximum (Q3 + 1.5 * IQR) and minimum (Q1 – 1.5 * IQR); and dots, outliers.

**Figure 2:**
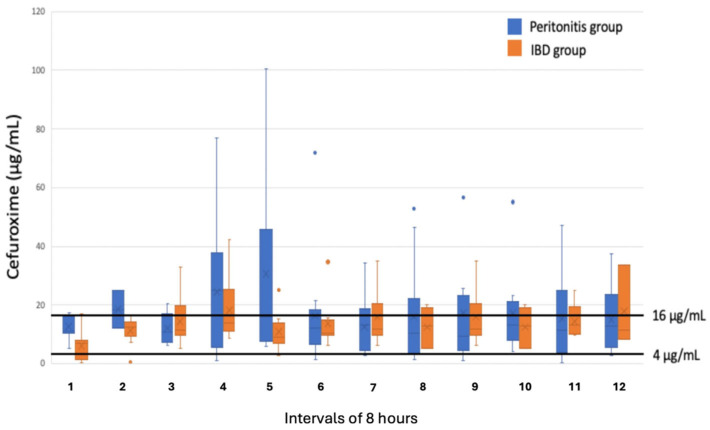
Box plots of the concentration of cefuroxime in microdialysates collected within 8 h from the peritoneal cavity in patients electively operated on for inflammatory bowel disease (IBD) and patients operated on for acute secondary peritonitis. Boxes indicate the interquartile rage (IQR) for Q1–Q3; bars, the maximum (Q3 + 1.5 * IQR) and minimum (Q1 – 1.5 * IQR); and dots, outliers.

There were no significant differences in the measured concentrations in the peritoneal fluid for either cefuroxime or metronidazole between the two groups (Figure 1 and Figure 2). A concentration of metronidazole ≥ 4 μg/mL was measured in all samples collected from the peritonitis group and in 99% of the samples collected in the IBD group. Regarding cefuroxime, for 88% of the samples in the peritonitis group and 93% of the samples in the IBD group, a concentration ≥ 4 μg/mL was measured. A concentration ≥ 16 μg/mL was only reached in 40% of the samples from the peritonitis group and in 23% from the IBD group.

In the peritonitis group, the interindividual as well as intraindividual variation in the concentrations measured was greater for both cefuroxime and metronidazole compared to in the IBD group (Table 2).

In the IBD group, the mean difference in concentration between participants 1–4 (sampling started 4 h after antibiotic administration) and participants 5–11 (sampling started immediately after antibiotic administration) was 1.5 for MTZ [−2.1 to 2.9] and 2.1 for CEF [−5 to 9.3].

## 3. Discussion

With the antibiotic regimen of cefuroxime and metronidazole used in this study, a concentration of metronidazole of ≥4 μg/mL, corresponding to the MIC value for most anaerobic strains, was measured in almost all collected microdialysates from both the IBD and peritonitis groups. Despite the relative long serum half-life time for metronidazole (6–10 h), no tendency of accumulation in the peritoneal fluid was found in any of the patients from the two groups.

For cefuroxime, a concentration of ≥4 μg/mL, corresponding to the MIC value for most Gram-negative strains, was reached in >87% of the samples in both groups. A concentration of ≥16 μg/mL, corresponding to the MIC value for mores more resistant bacterial strains, was measured in only 40% of the samples in the peritonitis group and 23% in the IBD group. In samples with a measured value below the MIC value, the true value might exceed the MIC value due to the missing correction for recovery rate. Given the small cohort size, this observation cannot be interpreted as evidence of clinical adequacy of the measured antibiotic exposure. With the relatively few patients included in this study, we could not investigate the clinical relevance of these findings. However, none of the patients in our study developed any clinical signs of surgical site infectious or other infectious complications during the postoperative period.

In addition, our study found that the sampling in relation to the administration of the antibiotics in the IBD group did not have any influence on the results. This is not surprising as the 8-h sampling interval corresponded to the interval of antibiotic administration.

Several different methods for measuring antibiotic concentration in different body liquids have been described [7]. The most used methods are liquid chromatography–mass spectrometry or high-pressure liquid chromatography. These methods offer high accuracy and are regarded as gold standards. Although liquid chromatography–mass spectrometry is more costly and complex, it offers the highest sensitivity and specificity. For clinical monitoring in larger scales, emerging techniques, including fluorescent probes or electrochemical sensors, may be useful, but none of these methods have been approved for intraperitoneal monitoring.

From our results, it is not possible to say anything about the fluctuations in the concentration over time within each sampling period of either antibiotic administered, but only about the cumulative concentration during a sampling period, which was equivalent to the time interval between the administration of the drugs. Sampling periods with shorter intervals might have given more information on the fluctuation in the concentration of the antibiotics in relation to drug administration, such as on the length of the time period where the value of the concentration was above that for the MIC (T > MIC), the area under the curve in relation to the MIC (AUC/MIC), and the maximum concentration obtained in relation to the MIC (C_max_/MIC). These parameters are traditionally used to evaluate the pharmacokinetics in serum but are more difficult to obtain in target organs, including the peritoneal fluid. Metronidazole is a concentration-dependent antibiotic, for which AUC/MIC is generally considered the most relevant pharmacodynamic target. In contrast, cefuroxime is a time-dependent antibiotic, where T > MIC is the most appropriate efficacy target. Our results focused exclusively on whether the measured concentrations exceed predefined MIC thresholds, which may oversimplify the pharmacodynamic interpretation. Thus, the clinical relevance of a given MIC value should be interpreted with caution [8].

To achieve the traditional pharmacokinetic parameters mentioned above, a sampling of microdialysates with much shorter intervals would be necessary. This would have required a considerably higher perfusion rate to achieve sufficient material for the concentration analysis. However, a higher perfusion rate compromises the relative recovery in microdialysis [9]. An important limitation of our study is that the recovery rate of the administered antibiotics was not analysed. An in vivo estimation of the relative recovery in our study would require retrodialysis before or after treatment completion, which was considered unethical to perform in our two patents groups. Previous in vitro studies have shown a high recovery rate of more than 75% for both cefuroxime and metronidazole using a microdialysis principle similar to that used in this study [10,11]. Other studies have shown no interference in recovery rate with a combined antibiotic administration [12]. To our knowledge, no other studies on recovery rate in peritoneal fluid have been published. Therefore, the true concentrations in our study might have been approximately 25% higher than the measured values.

The advantage of the microdialysis principle is its ability to measure the concentration of various administered compounds or drugs and their diffusion into the interstitial fluid of various tissue compartments where the drug is intended to elicit its effect. This has not been studied for the peritoneal fluid in humans, and one important finding was that the diffusion of both antibiotics investigated was similar in peritonitis compared to non-peritonitis, but with higher intra- and intervariability in the peritonitis group.

IBD patients were chosen as the control group because they receive standard postoperative antibiotic treatment for 3 days postoperatively due to an increased risk of surgical site infections, and because this therapeutic regimen is similar to that used for the treatment of secondary peritonitis at our institution. IBD patients had a lower coefficient of variation for both interindividual and between individuals for both metronidazole and cefuroxime compared to the group of patients with peritonitis. This might indicate that ongoing peritonitis may influence the penetration of antibiotics to the peritoneal cavity. The absence of statistically significant differences concentrations of the two antibiotics between the two groups must be interpreted with caution because of the low number of patients included in the two groups. Larger-scale studies are needed to confirm this.

Peritonitis was not graded in this study, which is another limitation. This limits a reliable interpretation of the observed higher intra- and interindividual variability in the peritonitis group, and any results indicating the degree of peritoneal inflammation influencing antibiotic penetration remain speculative. However, a reliable statistical analysis on possible correlation between the degree or extent of peritonitis would not have been possible with this low number of patients.

## 4. Material and Methods

### 4.1. Setting

This open, prospective observational single-centre study was conducted at the surgical department of Odense University Hospital in accordance with the Declaration of Helsinki and following approvals from the Regional Scientific Ethical Committees for Southern Denmark (ID: S-20130018 and ID: S-S-20190095) and the Danish Data Protection Agency (19/36273).

### 4.2. Participants

The inclusion criteria were as follows: patients undergoing emergency surgery for secondary peritonitis due to a perforation on the small intestine, colon, or rectum or elective surgery for IBD and age older than 18 years. The exclusion criteria were an eGFR < 30 mL/min/1.73 m^2^, known allergies to metronidazole or cefuroxime, disseminated cancer disease, postoperative vacuum-assisted closure, or peritonitis confined to less than two quadrants. Informed written consent was obtained from all participants prior to inclusion. A total of 11 patients operated on for inflammatory bowel disease and 7 patients operated on for secondary peritonitis were included.

### 4.3. Peritoneal Microdialysis

At the end of surgery and before closure of the abdomen, a CMA 62 microdialysis catheter (M Dialysis AB, Stockholm, Sweden) was placed into the peritoneal cavity through a transcutaneous approach outside the incision, with the tip of the catheter floating freely between intestinal loops. In patients with secondary peritonitis, the catheter was placed in the most severely affected quadrant. The catheter was perfused continuously with Perfusion Fluid (T1^®^, M Dialysis AB, Stockholm, Sweden) using a CMA 106 or 107 microdialysis syringe pump (M Dialysis AB, Stockholm, Sweden). The flow rate was set to 0.3 μL/min.

All patients received intravenous antibiotics with 3 g of cefuroxime (Zinacef^®^, Actavis, Gentofte, Denmark) and 1.5 g of metronidazole (Baxter A/S, Allerød, Denmark) during anaesthesia induction, and continued with 1.5 g cefuroxime and 500 mg metronidazole postoperatively at 8-h intervals for at least 3 days.

In the peritonitis group, dialysates were collected every 8 h starting immediately after the first antibiotic administration at 06.00 a.m. on the first postoperative day. For the IBD patients, sampling started 4 h (10.00 a.m.) after the first administration and every 8 h afterwards in the first 4 patients and in the last 7 patients immediately after the first administration (06.00 a.m.). All samples were immediately stored at −80 °C for later analysis.

### 4.4. Measuring the Concentrations of Cefuroxime and Metronidazole

#### 4.4.1. Preparation of Stock Solutions, Calibration Samples, and Quality Control Samples

For cefuroxime and metronidazole (USP 1098209 and USP 1442009, from Sigma-Aldrich, St. Louis, MO, USA), a 500 μg/mL stock solution in methanol was prepared. A working solution of 5.0 μg/mL was prepared by diluting the stock solution with Milli-Q^®^ water. Then, a seven-point calibration curve of 0.02, 0.1, 0.5, 1.0, 5.0, 10.0, and 20.0 μg/mL was constructed. QC samples were prepared from a 500 μg/mL methanolic stock solution and a 5.0 μg/mL aqueous working solution in low-, mid-, and high-level Milli-Q^®^ water (0.05, 3.75, and 11.0 μg/mL) through the appropriate dilution of the QC working solution.

Internal standard stock solution (1000 μg/mL in methanol) was prepared by adding appropriate amounts of Cefuroxime-d3 and Metronidazole-d4 (Toronto Research Chemicals, Vaughan, ON, Canada). An internal standard working solution (10 μg/mL) was prepared through the dilution of stock solution with methanol.

#### 4.4.2. Sample Preparation

A total of 12.5 μL of internal standard solution (10 μg/mL) was added to a 25 μL blank, calibrator QC, and patient sample, and 75 μL of acetonitrile was added to precipitate proteins. Then, 625 μL of 10 mM aqueous ammonium acetate with 5% methanol (*v*/*v* %) was added and mixed completely. Samples were centrifuged for 20 min. at 3750× *g*. After centrifugation, the clear supernatant was transferred to HPLC vials.

#### 4.4.3. Liquid Chromatography–Mass Spectrometry Analysis

Cefuroxime and metronidazole were separated on a Phenomenex Gemini C18, 50 × 3.0 mm, 3 μm column using gradient elution, mobile phase A (10 mM aqueous ammonium acetate with 5% methanol (*v*/*v* %)), and mobile phase B (10 mM ammonium acetate in methanol). The initial conditions were 97% A, which was maintained for 1.0 min, and then B was increased to 95% over 5 min and was maintained for 1.0 min. Then, the initial conditions were re-established within 0.3 min. The complete runtime was 10.0 min, and the retention times for cefuroxime and mitronidazole were 6.35 min and 5.65 min, respectively.

Heated electrospray at 250 °C was used for ionisation. Cefuroxime was detected by monitoring the transitions from *m*/*z* 423.6 to *m*/*z* 207.1 and *m*/*z* 423.6 to *m*/*z* 318.1. Metronidazole was detected by monitoring the transitions from *m*/*z* 172.0 to *m*/*z* 82.1 and *m*/*z* 172.0 to *m*/*z* 128.0.

Cefuroxime-d3 was detected by monitoring the transitions from *m*/*z* 426.6 to *m*/*z* 210.1 and *m*/*z* 426.6 to *m*/*z* 321.1. Metronidazole-d4 was detected by monitoring the transitions from *m*/*z* 176.0 to *m*/*z* 82.1 and *m*/*z* 128.0, respectively.

### 4.5. Statistics

No power calculation was performed as this was an explorative observational study.

We registered the number of samples where the MIC in the collected dialysates reached 4 μg/mL for metronidazole and 4 and 16 μg/mL for cefuroxime, respectively.

We compared the mean concentration between the two patient groups and between the two different collection methods in relation to antibiotic administration in the IBD group using linear mixed models. With this analysis the variability in baseline measures (random intercepts) and variability in the relationship between predictor and outcome variables (random slopes) across groups was obtained. The variations in concentrations within each participant and variations between group members is reported as coefficient of variation (CV). This method assumes normality of residuals as well as random effects, which were investigated by quantile–quantile plots, but remains aware of the limited sample size making deviations from model assumptions challenging to detect.

## 5. Conclusions

In conclusion, peritoneal microdialysis is a feasible method of studying the diffusion of antibiotics into the peritoneal cavity. A method that measures the accumulative concentration of the antibiotics in the peritoneal fluid corresponding to the drug administration interval has the potential to provide important information to consider alongside traditional pharmacodynamic parameters and may be relevant to achieving an optimal therapeutic effect.

## Figures and Tables

**Table 1:** Basic demographic characteristics of the individuals from each group. Body mass index (BMI), SD = standard deviation.

Group	Gender (Female/Male)	BMI (Mean/SD)	Age (Mean/SD)
Peritonitis	5/2	25.0/2.6	62.9/10.6
Inflammatory bowel disease	4/7	24.3/4.3	46.6/11.1

**Table 2:** Intra- and interindividual coefficient of variation (CV) in the measurements of metronidazole (MTZ) and cefuroxime (CEF) concentrations in the two groups and with a 96% confidence interval (CI).

Group		MTZ Concentration		CEF Concentrations	
		CV%	[95% CI]	CV%	[95% CI]
Secondary peritonitis	Interindividual	63	[21–185]	189	[64–561]
	Intraindividual	36	[28–46]	116	[90–150]
Inflammatory bowel disease	Interindividual	21	[16–30]	25	[18–35]
	Intraindividual	5.5	[1.1–30]	4.8	[1.0–23]

## Data Availability

Data are contained within the article.
